# Supplementary material for: Southern Ocean CO2 outgassing and nutrient load reduced by a well-ventilated glacial North Pacific
Source: Nat Commun. 2025 Sep 17;16:8279. doi: 10.1038/s41467-025-63774-8 (PMC12443982; doi:10.1038/s41467-025-63774-8)
Supplement: Supplementary file 1 — Supplementary Information [file 41467_2025_63774_MOESM1_ESM.pdf]

Supporting Information for

Southern Ocean CO_2 outgassing and nutrient load reduced by a well-ventilated glacial North Pacific

Madison G. Shankle[1]*; Graeme A. MacGilchrist[1]; William R. Gray[2]; Casimir de Lavergne[3]; Laurie C. Menviel[4]; Andrea Burke[1]; James W. B. Rae[1]

[1] School of Earth and Environmental Sciences, University of St Andrews, St Andrews, KY16 9TS, United Kingdom

[2] Laboratoire des Sciences du Climat et de l'Environnement (LSCE/IPSL), CEA-CNRS-UVSQ, Université Paris-Saclay, Gif-sur-Yvette, 91190, France

[3] LOCEAN Laboratory, Sorbonne Université-CNRS-IRD-MNHN, Paris, 75005, France

[4] Climate Change Research Centre, The Australian Centre for Excellence in Antarctic Science, University of New South Wales, Sydney, NSW 2052, Australia

*Corresponding Author: Madison G. Shankle.

Email: mgs23@st-andrews.ac.uk

Address: School of Earth and Environmental Sciences, University of St Andrews, Bute Building, Queen's Terrace, St Andrews, KY16 9TS, Scotland, United Kingdom

Phone number: +44 (0)1334 46 3940

Contents:

Supplementary Figure 1

Supplementary Figure 2

Supplementary Figure 3

Supplementary Figure 4

Supplementary Figure 5

Supplementary Figure 6

Supplementary Figure 7

Supplementary Figure 8

Supplementary Figure 9

Supplementary Table 1

Supplementary Table 2

Supplementary References

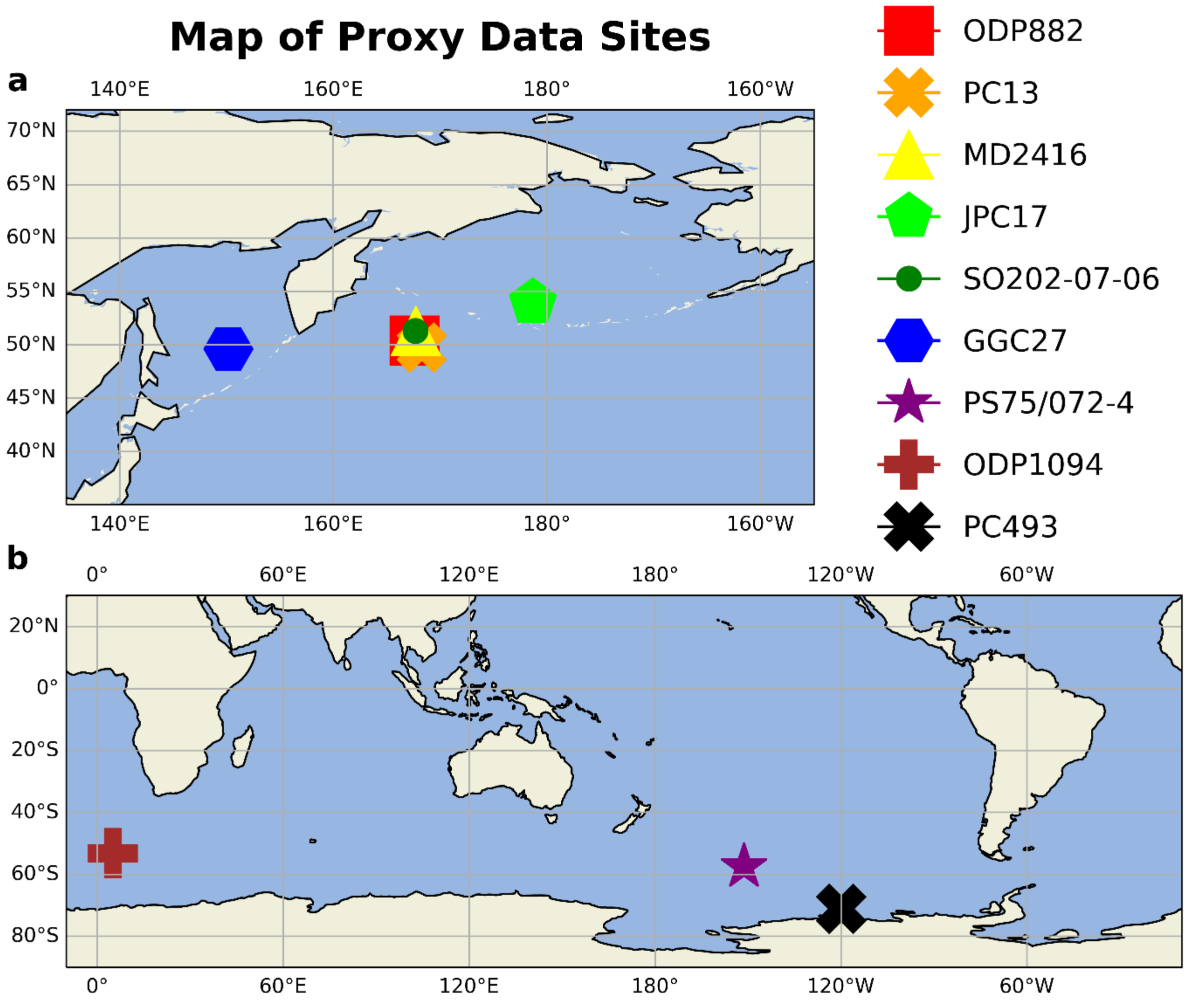

Supplementary Figure 1. **Maps depict the locations of marine sediment core sites providing the proxy data in Figure 2.** The proxy data presented in Figure 2 comes from a range of sites in the **(a)** North Pacific and **(b)** Southern Ocean.

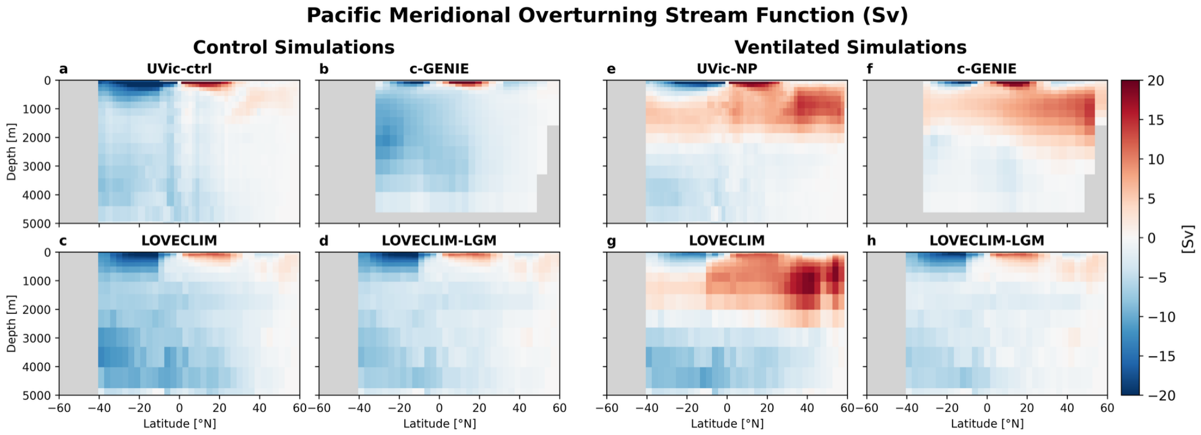

Supplementary Figure 2. **Ventilation and overturning developing in the North Pacific is seen in various Earth System Models in response to North Atlantic freshwater forcing.** Meridional overturning stream function (Sv) in intermediate-complexity Earth System Models run under glacial-like boundary conditions either with **(e-h)** or without **(a-d)** freshwater perturbations to induce overturning in the North Pacific. Each panel depicts the average over the last 10 years of simulation, except in the case of c-GENIE which takes snapshots at the final year of each simulation. For a quantification of the maximum overturning stream function in each case, see Supplementary Table 1. For the details of the models and their forcing, see Supplementary Table 2.

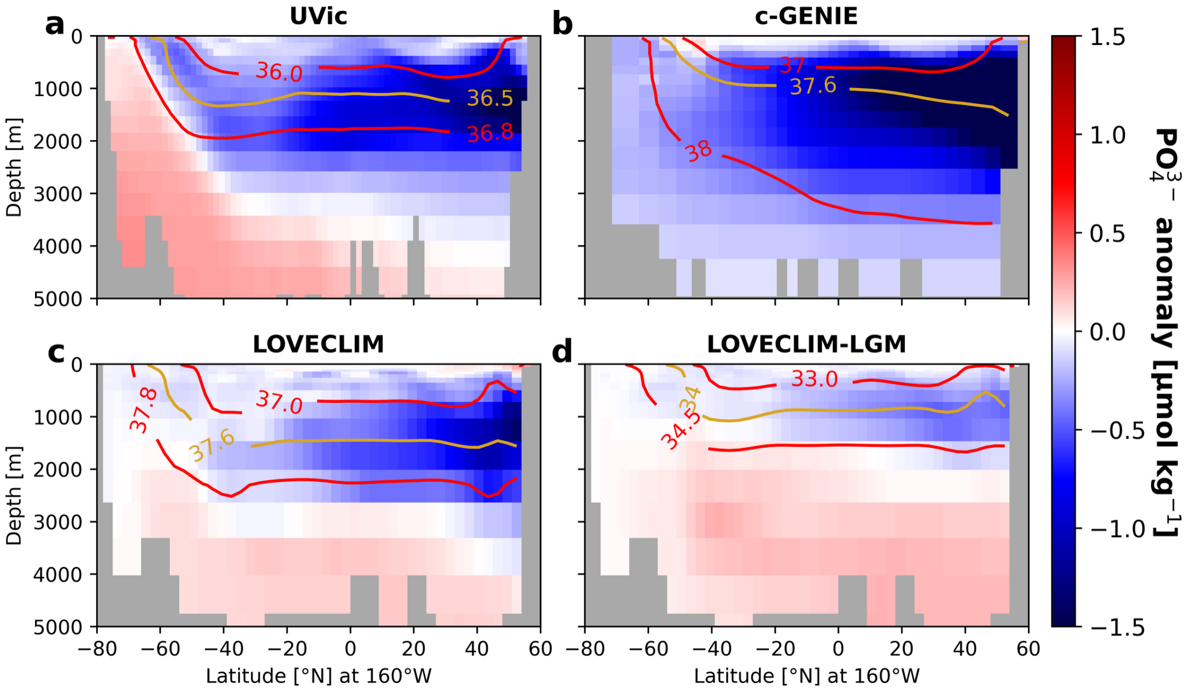

Supplementary Figure 3. **Earth system models simulating a well-ventilated glacial North Pacific show negative anomalies in phosphate (PO_4^{3-}) (µmol kg^{-1}) throughout the basin.** Phosphate anomalies along 160°W resulting from a well-ventilated glacial North Pacific in the **(a)** UVic[1], **(b)** cGENIE[2], **(c, d)** and LOVECLIM[1,3] Earth System Models, showing the same pattern as potential *p*CO_2 (PCO_2) anomalies depicted in Figure 3. Anomalies compare averages over the last 10 years of each simulation, except in the case of c-GENIE which takes snapshots at the final year of each simulation. Red and yellow contours show isolines of σ_2 (potential density referenced to 2000 dbar, over the same time periods as the PO_4^{3-} output) from the perturbed simulation in each case, meant to approximate the neutral surfaces along which these waters are expected to flow.

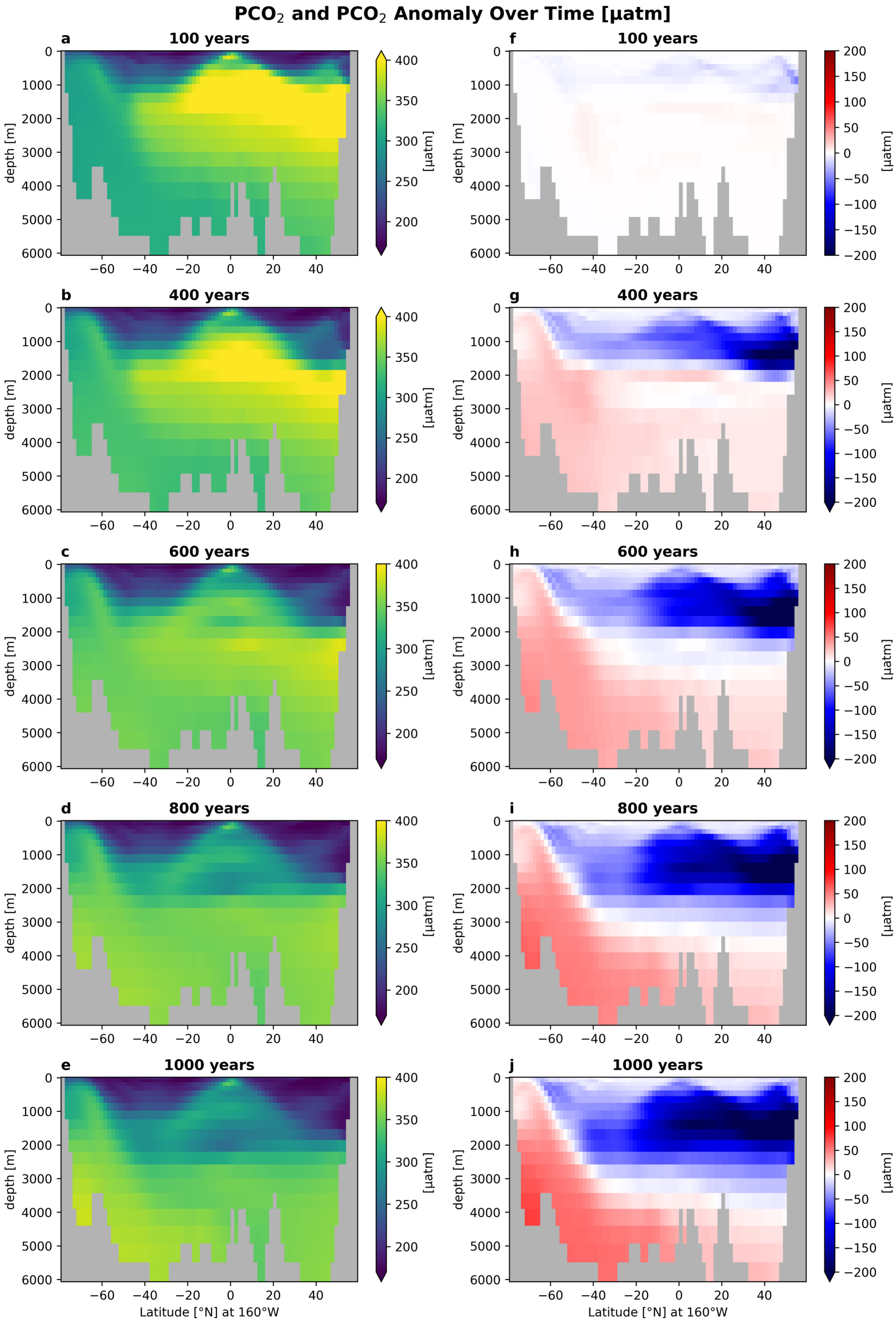

Supplementary Figure 4. **The time-evolution of potential pCO_2 (PCO_2) (µatm) in UVic-NP shows carbon accumulating in deep waters and negative anomalies developing from the north. (a-e)** Absolute PCO_2 (see Methods) (µatm) along 160°W over the 1000 years of the UVic-NP simulation. **(f-j)** PCO_2 anomaly relative to UVic-ctrl (µatm) over the same time. Anomalies compare averages over the last 10 years of each simulation.

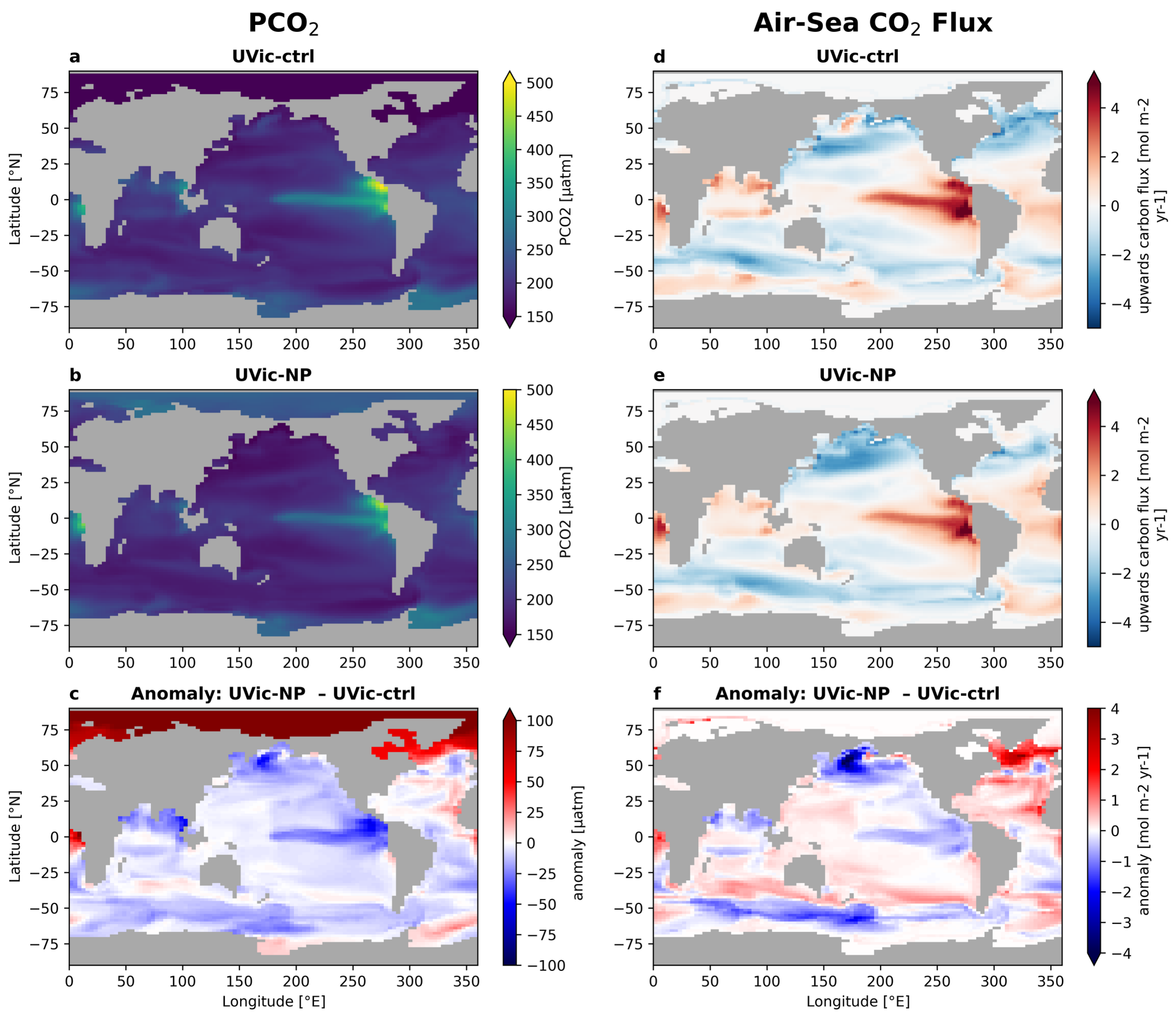

Supplementary Figure 5. **Negative anomalies in potential pCO_2 (PCO_2) pervade the Indian-Pacific basin and Southern Ocean, driving negative anomalies in air-sea CO_2 flux in key outgassing regions.** PCO_2 (see Methods) (µatm) **(a, b)** and air-sea CO_2 flux (mol m^{-2} yr^{-1}) **(d, e)** in UVic-ctrl **(a, d)**, UVic-NP **(b, e)**, and as anomalies (UVic-NP – UVic-ctrl) **(c, f)**. Anomalies compare averages over the last 10 years of each simulation. Negative flux anomalies indicate reduced CO_2 outgassing or enhanced CO_2 uptake. Regions of positive outgassing anomalies in the Indian-Pacific basin correspond with regions of weakly-negative or zero PCO_2 anomaly, indicating they are driven by the slightly decreased atmospheric pCO_2 in UVic-NP (~185 µatm) relative to UVic-ctrl (~191 µatm). Positive flux anomalies in the North Atlantic are expected to be a result of the freshwater forcing applied in this region in the UVic-NP simulation, again unrelated to North Pacific ventilation. The addition of freshwater to the Atlantic dilutes surface

alkalinity, contributing to positive PCO_2 and flux anomalies; stronger stratification resulting from the freshwater input may also contribute to reduced surface alkalinity. The sluggish overturning and greater stratification also result in poorer ventilation of North Atlantic sub-surface waters, which accumulate more remineralized carbon with which to supply outgassing. Finally, the reduced atmospheric pCO_2 (-6 ppm) between the simulations is expected to drive some of the anomalous outgassing signal, especially in regions where changes in surface PCO_2 are minimal (compare regions of low-to-no surface PCO_2 anomaly **(c)** to the more pervasive flux anomalies in **(f)**). Notably, despite the Atlantic basin accumulating carbon due to a weakened AMOC and contributing positive PCO_2 anomalies to the Southern Ocean surface, the net effect in the Southern Ocean remains a reduction in surface carbon and nutrients, fueled by the negative anomalies supplied from the Pacific. This highlights the dominant role played by Pacific waters in shaping surface Southern Ocean biogeochemistry.

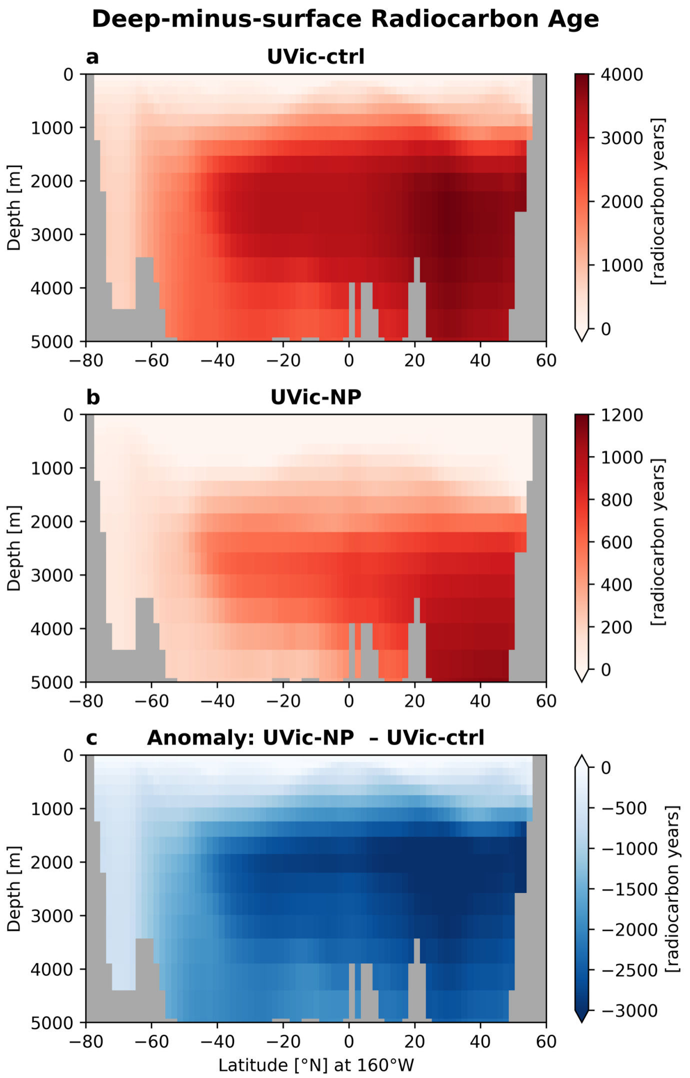

Supplementary Figure 6. **North Pacific ventilation is evidenced by strong reductions in deep-minus-surface radiocarbon age.** Deep-minus-surface radiocarbon years (equivalent to benthic-planktic foraminifera radiocarbon ages) in **(a)** UVic-ctrl and **(b)** UVic-NP, with each grid cell's radiocarbon years value having subtracted from it the radiocarbon years value of the surface cell at its same latitude and longitude. **(c)** Radiocarbon years anomaly (UVic-NP – UVic-ctrl), with negative anomalies indicating younger (less radiocarbon-depleted) better-ventilated waters. Output in each panel represents averages over the last 10 years of simulation. Note, while deep-minus-surface radiocarbon years is equivalent to benthic-plankton foraminifera radiocarbon ages, it is not possible to directly compare this model to glacial radiocarbon proxy data because of how atmospheric ^{14}C was allowed to vary in the model. While the strong

ventilation in UVic may over-estimate the age anomaly, this figure is still indicative of the changes expected from North Pacific ventilation; large changes are expected with ventilation replacing what was previously the global ocean’s oldest waters with now very young waters.

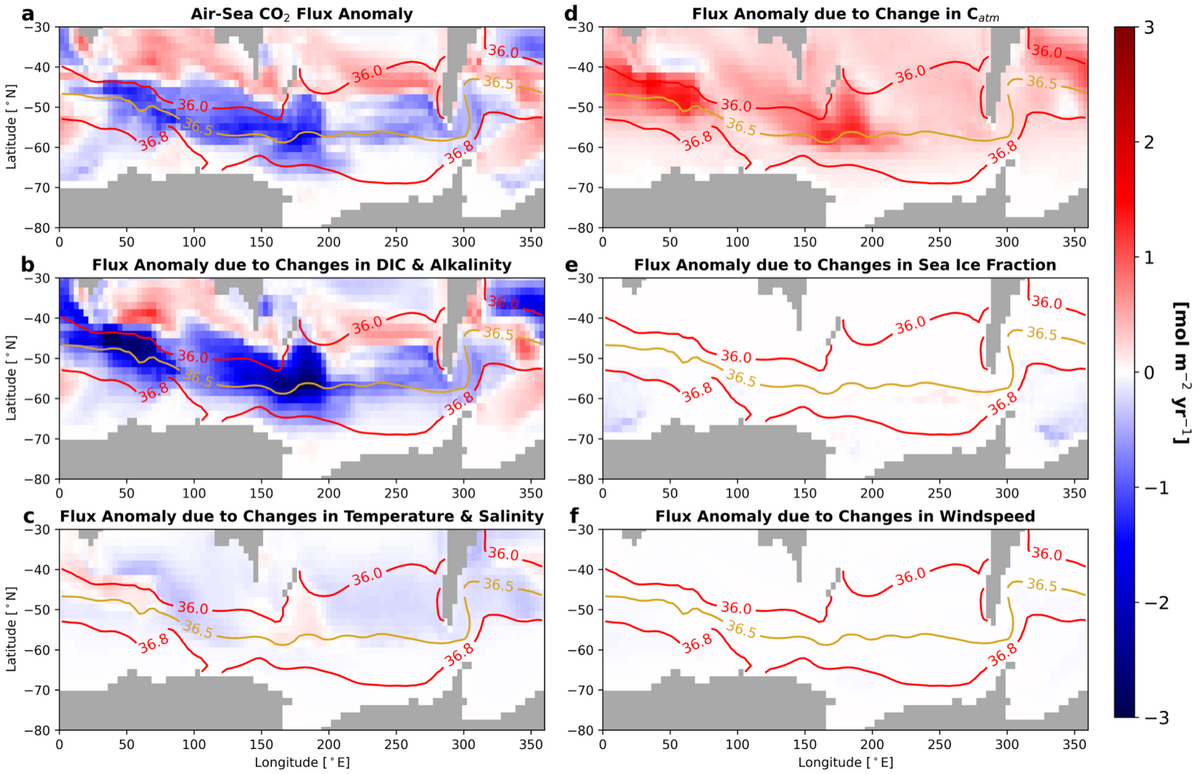

Supplementary Figure 7. **Southern Ocean air-sea CO_2 flux anomaly is dominated by changes in the water's alkalinity and dissolved inorganic carbon (DIC) content. (a)** Air-sea CO_2 flux anomaly (mol m^{-2} yr^{-1}) between the control and perturbed simulations (UVic-NP – UVic-ctrl), with negative values indicating reduced CO_2 outgassing or enhanced CO_2 uptake. **(b-f)** Air-sea CO_2 flux anomaly attributable to changes in the stated variable alone. Flux is first computed with the variable of interest as simulated by the UVic-NP simulation and fields for all other variables as simulated by the UVic-ctrl simulation. The resulting flux field then has subtracted from it the flux field obtained from the UVic-ctrl simulation, computed using UVic-ctrl fields for all variables. The resulting anomaly thus gives the anomaly in flux due only to changes in the variable of interest from UVic-ctrl to UVic-NP. Note that **(d)** depicts the CO_2 flux anomaly due to changes both in atmospheric pCO_2 and in CO_2 solubility (a function of temperature and salinity changes between UVic-ctrl and UVic-NP); changes in atmospheric pCO_2 dominate the positive-anomaly signal, though the isolated regions of greater anomalies are attributed to temperature changes. Changes in **(e)** fractional sea ice cover and **(f)** wind speed also play a negligible role in the reported CO_2 flux anomaly **(a)**. Anomalies compare averages over the last 10 years of each

simulation. Red and yellow contours show isolines of σ_2 (potential density referenced to 2000 dbar) from the perturbed simulation (averaged over the last 10 years of the UVic-NP simulation), meant to approximate the neutral surfaces along which these waters are expected to flow. σ_2 is plotted at 500m depth so as to avoid any influence of mixed layer processes.

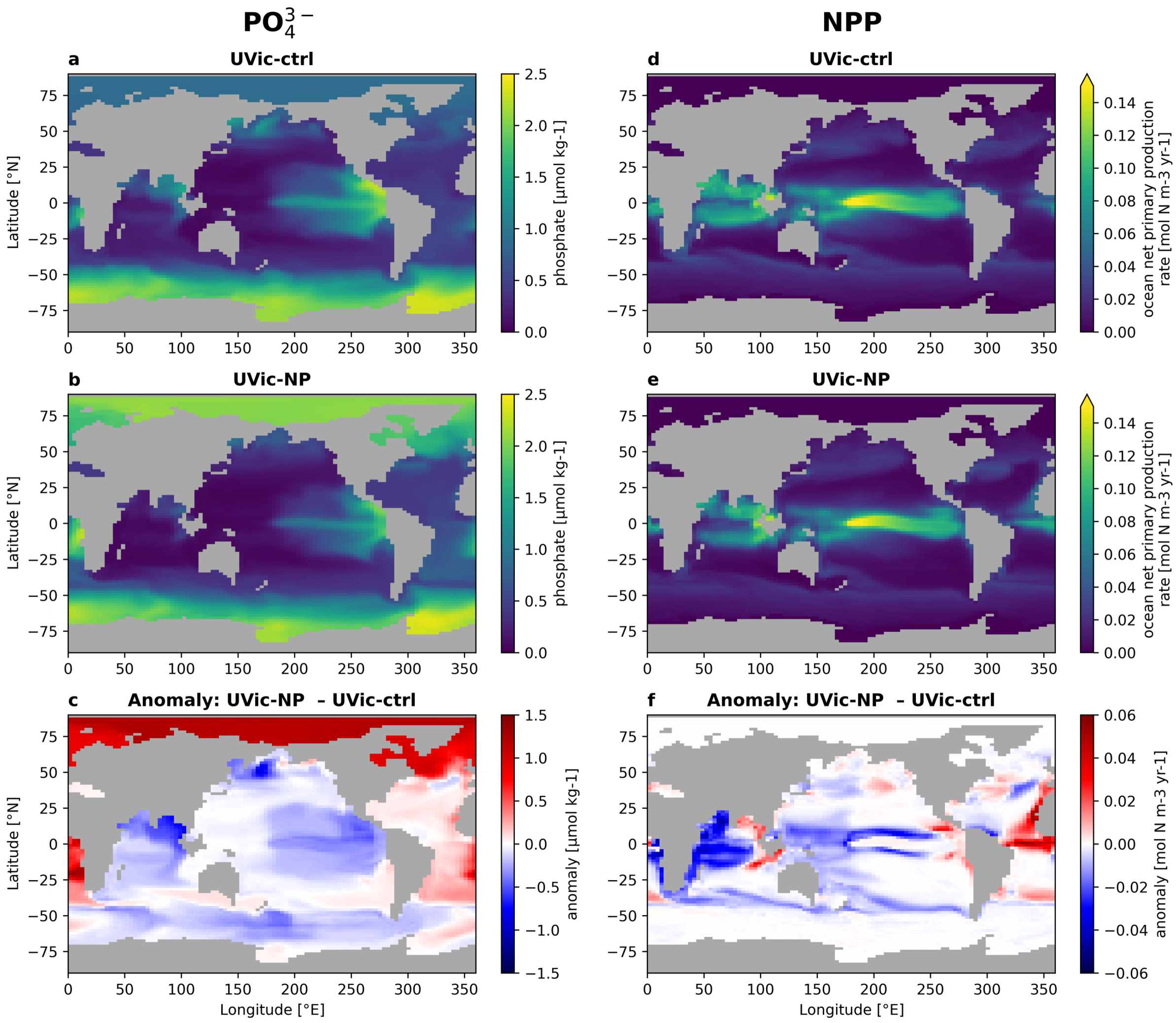

Supplementary Figure 8. **Negative anomalies in surface phosphate (PO_4^{3-}) concentration pervade the Indian-Pacific basin and Southern Ocean, with net primary production (NPP) following suit.** Surface phosphate (PO_4^{3-}) concentration (µmol kg^{-1}) **(a, b)** and net primary production rates (NPP, mol N m^{-3} yr^{-1}, describing the rate of change of the molar nitrate concentration in a model grid cell driven by NPP) **(d, e)** in UVic-ctrl **(a, d)**, UVic-NP **(b, e)**, and as anomalies (UVic-NP – UVic-ctrl) **(c, f)**. Anomalies compare averages over the last 10 years of each simulation. Negative phosphate anomalies across the Indian-Pacific basin in response to North Pacific ventilation **(c)** drive corresponding reductions in NPP **(f)** (except in regions of already low biological production, such as subtropical gyres and the Southern Ocean, which see little or no anomalies in NPP).

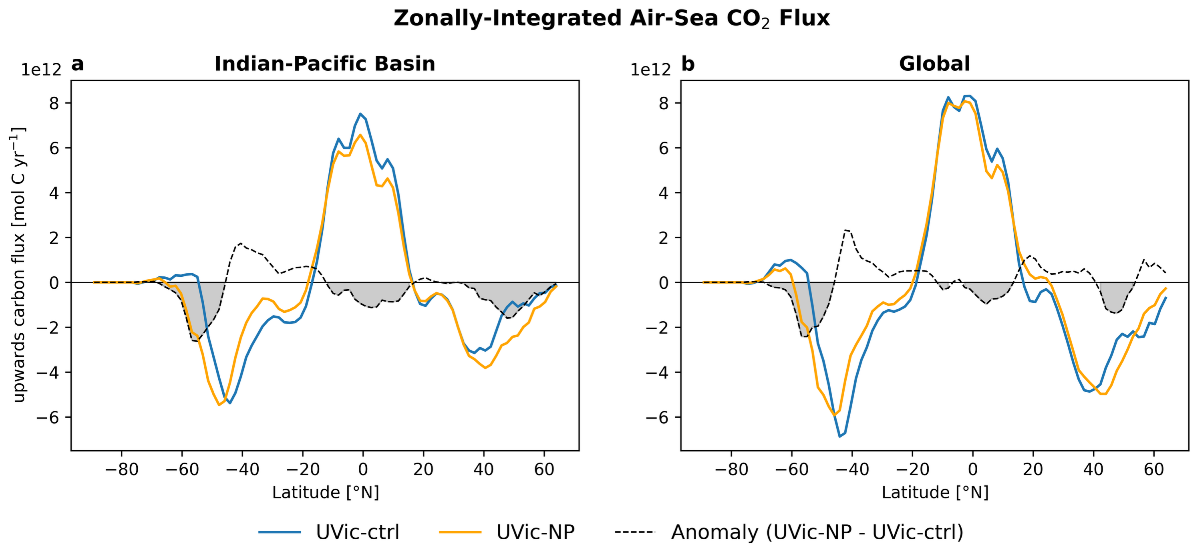

Supplementary Figure 9. **North Pacific ventilation reduces outgassing in high latitude (>40°N/S) and equatorial (±20°) regions.** Zonally-integrated upwards CO_2 flux (mol C yr^{-1}, with positive values denoting flux out of the ocean) as a function of latitude, integrated across the **(a)** Indian-Pacific basin and **(b)** entire globe. Flux in UVic-ctrl and UVic-NP are shown in blue and orange, respectively, with the anomaly (UVic-NP – UVic-ctrl) plotted in the dashed black line. Negative values of the anomaly (grey shading) indicate either reduced outgassing or enhanced ingassing. In both UVic-ctrl and UVic-NP, the ocean is equilibrated with the atmosphere, with the land, ocean, and atmospheric carbon inventories all having roughly equilibrated (not shown). The zonal integral across the Indian-Pacific basin was computed by masking the data to only this basin using CMIP6 basin codes available through the "cmip_basins" Python package (https://github.com/jkrasting/cmip_basins).

Maximum Meridional Overturning Stream Function (Sv), North of 31 °N				
	UVic	c-GENIE	LOVECLIM	LOVECLIM-LGM
N. Pacific, Control (“UVic-ctrl”)	3.2	0.8	2.8	2.8
N. Pacific, Ventilated (“UVic-NP”)	14.8	14.4	19.0	4.1
N. Pacific Anomaly (Ventilated – Control)	*+11.6*	*+13*	*+16.2*	*+1.3*
N. Atlantic, Control	14.2	14.2	20.9	21.8
N. Atlantic, Ventilated	1.3	0.1	1.8	13.8
N. Atlantic Anomaly (Ventilated – Control)	*-12.9*	*-14.1*	*-19.1*	*-8.0*

Supplementary Table 1. **Model comparison of North Pacific and North Atlantic overturning strengths.** Maximum of the meridional overturning stream function north of 31°N across the four models depicted in Figure 3. The cut off of 31°N removes the influence of subtropical gyre circulation across all four models, but the results are otherwise insensitive to choice of latitude. Notably, despite the wide range in magnitudes of overturning anomalies, all models see a reduction of Pacific mid-depth carbon and nutrients in response to North Pacific ventilation (Fig. 3, Supplementary Fig. 3). Note also that in the ventilated LOVECLIM-LGM simulation, Pacific overturning expands in extent more so than magnitude over the control (see Supplementary Fig. 2). It should also be noted that, while the LOVECLIM-LGM simulation is presented as an estimate of overturning strengths at the LGM, the magnitude of glacial overturning (Atlantic and Pacific) remains uncertain. For example, simulations in c-GENIE find an overturning of ~8 Sv presents the best model-data fit for LGM proxy data[2], contrasting with the ~4 Sv estimate in LOVECLIM-LGM. Results from LOVECLIM-LGM (e.g., Fig 3d and Supp. Fig. 3d) should therefore be taken as indicative rather than definitive.

	UVic	c-GENIE	LOVECLIM	LOVECLIM-LGM
Reference	Menviel et al., 2014	Rae et al., 2020	Same as UVic	Menviel et al., 2017
Resolution (latitude x longitude, depth layers)	1.8° x 3.6°, 20 depth layers	5° x 10°, 16 depth levels	3° x 3°, 20 depth layers	3° x 3°, 20 depth layers
Ocean Component	MOM v2	c-GENIE (a frictional geostrophic 3D ocean model)	CLIO	CLIO
Boundary Conditions	LGM-like (Last Glacial Maximum, ~21 kaBP) for glacial topography and albedo, orbital parameters, and atmospheric CO_2 (192 ppmv)	Glacial-like for radiative forcing consistent with major greenhouse gas concentrations (CO_2, CH_4, N_2O), planetary albedo, increased average ocean salinity, and atmospheric CO_2 (278 ppmv)	LGM-like (Last Glacial Maximum, ~21 kaBP) for glacial topography, planetary albedo, orbital parameters, and atmospheric CO_2 (191.85 ppmv)	LGM-like (first equilibrated under 35 kaBP boundary conditions and run transiently to 20 kaBP) glacial topography and albedo, orbital parameters, and atmospheric CO_2 (190 ppmv)
Forcing Style	N. Atlantic freshwater hosing	Reduced prescribed atmospheric freshwater flux from N. Atlantic to N. Pacific	N. Atlantic freshwater hosing	N. Atlantic freshwater hosing
Forcing Details	0.1 Sv freshwater for 1000 years into N. Atlantic (55-10°W, 50-65°N)	-0.28 Sv of freshwater forcing into N. Pacific over 5000 years	Same as UVic	0.05 Sv of freshwater forcing into N. Atlantic over 4000 years
Simulation Run Time	1000 years	5000 years	Same as UVic	4000 years
Ventilation Strength (maximum overturning in N. Pacific, Sv)	14.8	14.4	14.4	3.7

Supplementary Table 2. **The output depicted in Figure 3 comes from glacial-like simulations of various intermediate-complexity Earth System Models spanning varying configurations, resolutions, forcings, etc.** All models depicted in Figure 3 were run under glacial-like boundary conditions and induced North Pacific ventilation and enhanced North Pacific Intermediate Water (NPIW) formation by manipulating freshwater forcing. In c-GENIE, the prescribed transfer of atmospheric freshwater from the Atlantic to the Pacific was reduced. In all other models, Atlantic overturning was suppressed by adding meltwater into the North Atlantic. Resulting oceanic and atmospheric teleconnections then led to enhanced NPIW formation. Beyond this, the models differ slightly in their details, which are summarized here. References: Menviel et al. (2014)[1], Menviel et al. (2017)[3], and Rae et al. (2020)[2].

Supplementary References

1. Menviel, L., England, M. H., Meissner, K. J., Mouchet, A. & Yu, J. Atlantic-Pacific seesaw and its role in outgassing CO2 during Heinrich events. *Paleoceanography* **29**, 58–70 (2014).

2. Rae, J. W. B. *et al.* Overturning circulation, nutrient limitation, and warming in the Glacial North Pacific. *Sci Adv* **6**, eabd1654 (2020).

3. Menviel, L. *et al.* Poorly ventilated deep ocean at the Last Glacial Maximum inferred from carbon isotopes: A data-model comparison study. *Paleoceanography* **32**, 2–17 (2017).
